# The impact of landscape complexity and composition on honey bee visual learning

**DOI:** 10.1242/jeb.250057

**Published:** 2025-07-04

**Authors:** Georgina Hollands, Jake L. Snaddon, Philip L. Newland, Suleiman M. Sharkh

**Affiliations:** ^1^ Mechatronics Research Group, Mechanical Engineering, University of Southampton, Highfield Campus, Southampton SO17 1BJ, UK; ^2^ School of Geography and Environmental Science, Highfield Campus, University of Southampton, Southampton SO17 1BJ, UK; ^3^ School of Biological Sciences, University of Southampton, Highfield Campus, Southampton SO17 1BJ, UK

**Keywords:** Landscape, Learning, Honey bee, *Apis*, Apidae

## Abstract

Over the past few decades there has been an overall decline in the number of pollinators, including wild bees, partly due to stress factors such as the availability of food resources, nest site availability and pesticide usage. Managed honey bees have also been negatively impacted in certain regions, such as the USA. One of the major stress factors facing bees currently is land use change, where natural landscapes are decreasing and often converted to either agricultural or urban land. Here, we assess directly the link between landscape diversity, edge density and honey bee learning, by analysing how honey bee visual learning ability varies across different landscapes, using a field-adapted version of the proboscis extension response. It was previously thought that honey bees from hives based in different landscapes may vary in visual learning abilities because of their different experiences and neural plasticity. Thus, bees that have experience in more complex learning environments may do better in learning tasks. To test this, bees were taught to associate a coloured yellow paper strip with a positive sugar reward and a blue coloured strip with a negative salt reward. Results showed that as edge density increased in the landscape, visual learning in bees reduced, and when landscape diversity increased, so did learning. This is important as bees must learn foraging routes, find profitable flowers and develop spatial maps, as well as recognise intruders. If their cognitive abilities are reduced and they are unable to carry out these tasks, this will be detrimental for the continuous development of the colony.

## Teaser

**Summary:**. Honey bees from diverse landscapes show better learning, while those from high edge density areas perform worse, highlighting the importance of landscape diversity for bee foraging and navigation.

## INTRODUCTION

The stability of managed honey bee populations varies across the globe, and while overall honey bee stocks increased by approximately 45% between 1961 and 2008, owing to an increase in the number of hives in countries such as China and Argentina (Goulson et al., 2015), as well as an increase of 5.5% between 2017 and 2018 in Europe (https://ec.europa.eu/agriculture/honey_en; accessed 09 July 2019), there have also been widespread reports of declines (Goulson et al., 2015). In North America the number of managed honey bee colonies was reported to have experienced a loss of 43% between 2019 and 2020 (Insolia et al., 2022). Numerous causal factors for such declines have been identified, including both socioeconomic, such as a decline in beekeeper numbers (Potts et al., 2010b), and environmental reasons, including stressors such as pathogens, pesticide exposure (Cappellari et al., 2024; Hisamoto et al., 2024), air pollution (Margaoan et al., 2025), and loss of foraging and nesting sites through habitat degradation (Potts et al., 2010a). While few of these stressors can be considered novel, their increased intensity over the past decade has led to worsening effects on both managed and wild bees (Klein et al., 2017). Many of these stressors can be linked to a changing landscape, particularly the intensification and expansion of agriculture (Laurance et al., 2014; O'Neal and Hall, 2024).

Land use change and associated increase of agricultural and urban land, along with an associated decline of natural habitat, is thought to be one of the major contributors to the decline of bee numbers (Otto et al., 2016; Potts et al., 2010a). The effects of changes in land use impact resource availability and have consequential impacts on bee diet, nesting availability and pesticide exposure (Goulson et al., 2015). The effects, both in terms of landscape composition (Garibaldi et al., 2013; Kennedy et al., 2013; Ricketts et al., 2008) and landscape configuration (Lonsdorf et al., 2009; Winfree et al., 2008), have largely been studied in terms of how they affect wild bee abundances and diversity (Du Clos et al., 2020). Few studies have, however, investigated how differences in landscape composition and configuration could affect bee behaviour, and even fewer have investigated how differences in landscape composition/configuration directly impact the ability of honey bees to learn. Menzel et al. (2019) compared honey bee flight in a featureless landscape and one rich in close and distant landmarks; flight routes and timings were studied, to infer learning. In their study, three different forms of learning were analysed: learning during orientation flight, learning during training to a feeding site and learning during homing flights. They found that bees used elongated ground structures, such as field boundaries separating two pastures, and hedgerows to orientate themselves. In a later study, landmark knowledge was found to be able to over-ride optic flow in honeybee waggle dance distance estimation (Menzel and Galizia, 2024). Sardar and Rehman (2023) similarly found that the honey bee experiences significantly lower flight path deviations when visual landmarks were in an altered state in comparison to an altered one. Furthermore, Degen et al. (2016) showed that bees learn the landscape and site structures during long-range orientation flights, leading to faster homing from explored areas. When bees were displaced before carrying out their first orientation flights, they were not able to find their way back to the hive. Bees are not the only insect species where landmarks have been shown to be important in navigation. The digger wasp (*Philanthus triangulum*), for example, has been shown to use landmarks to orientate back to their nest. In ground-breaking studies Tinbergen (1953) showed that by displacing pinecones around a wasps nest, a returning wasp returned to the pine cones rather than to the nest entrance itself. Desert ants (*Ocymyrmex robustior*) take ‘snapshots’ of their nest entrance whilst carrying out learning walks where landmarks were shown to be particularly important in a featureless environment (Müller and Wehner, 2010).

Until recently, the majority of studies investigating bee learning have been carried out in the laboratory and on honey bees that were either wild individuals brought into captivity or laboratory reared (Muth et al., 2018). Studying cognitive traits in the field have previously proven to be problematic because of numerous confounding variables and little control, and thus carefully designed experiments are needed to measure variation between colonies (Morand-Ferron et al., 2015; Muth et al., 2018; Rowe and Healy, 2014). Muth et al. (2018) developed a protocol called a ‘free-moving proboscis extension response (FMPER)’ where wild honeybees and the yellow face bumblebee (*Bombus vosnesenski*), were successfully taught to associate colour with both positive and negative rewards. This method has the potential to be used in the field with managed and wild bee species with limited equipment and costs (Muth and Leonard, 2019).

To our knowledge, no previous studies have been carried out investigating directly the effects of landscape complexity and configuration on honey bee learning. This study aims to investigate how landscape configuration affects honey bee (*Apis mellifera*) visual learning using the FMPER across 12 colonies from different apiaries across southern England. The ability for bees to learn was compared across a gradient of landscapes, ranging from low to high complexities in terms of configuration, and changes in landscape composition were measured.

## MATERIALS AND METHODS

### Study area

**Fig. 1.**
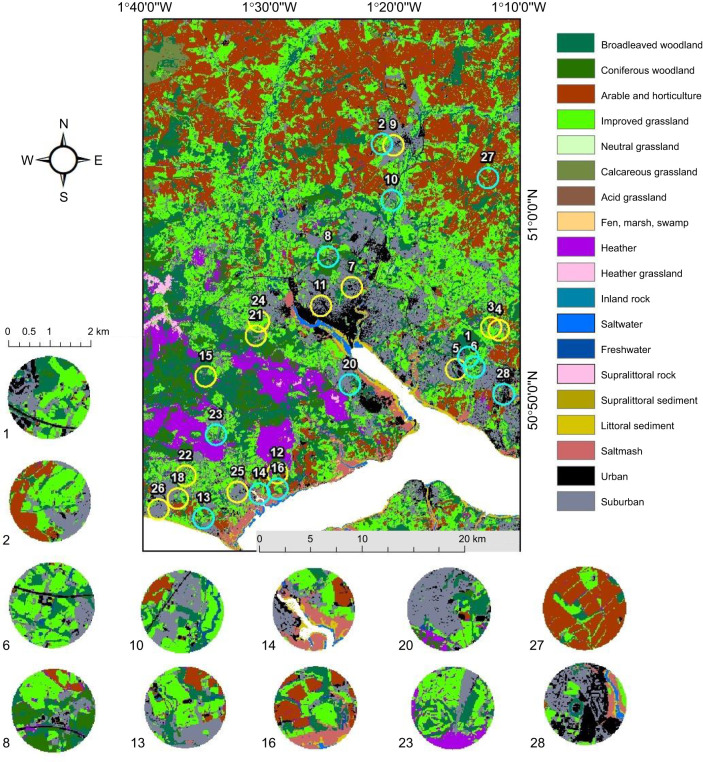
**Twenty-six apiaries located across southern England identified for this study.** The 12 apiaries used in this study are indicated by a blue circle, which represents the 1 km buffer zone around each hive. Closeups are displays of these sites. Yellow circles represent apiaries that were not chosen. Habitat types were obtained at a 25 m resolution from images collected by the Landsat 5 Thematic Mapper, from EDINA Digimap.

Twenty-six potential honey bee (*Apis mellifera* Linnaeus 1758) apiaries were identified from across southern Hampshire, UK. The landscape was dominated by improved grassland, coniferous woodland, arable and horticultural land as well as urban/suburban habitat (Fig. 1). Data collection occurred between 6 May and the 20 July 2021, where the average daily maximum temperatures ranged from 15 to 22°C (data from Weatherspark). Across Southampton 103.6 mm of rainfall occurred in May, 108.5 mm in June and 110.7 mm in July (data from Weatherspark).

From the 26 potential apiaries, 12 suitable sites were selected through a systematic process based on different selection criteria, including landscape composition, distances between sites, hive duration and hive health. Every hive owner was asked about the history of their hives, this included its age, queen history, instances of parasites such as *Varroa* mite and honey production. Only well-established hives with no history of disease/parasites with a stable queen were deemed suitable. Replication was carried out at the landscape level, each hive was tested three times, with only one hive tested per site. Hives across sites were tested in parallel and there was no more than 2 weeks between consecutive tests at each hive. This was because while hive replication contributes to the precision of each data point, increasing the number of landscapes increases the number of data points and therefore contributes to the primary objective of understanding how landscape heterogeneity impacts honey bee learning (Fahrig et al., 2011).

### Landscape metrics

Landscape metrics were based on the 2019 EDINA land cover map at a 25 m resolution, which used data from the Landsat 5 Thematic Mapper (EDINA Digimap). Land cover was split into 21 different categories (Fig. 1) based on a classification described by the Centre for Ecology and Hydrology (https://www.ceh.ac.uk/data/ukceh-land-cover-maps).

The landscape metrics were classified for buffers of 1 km radius surrounding each apiary. A 1 km scale was chosen as it represented the environment that the bees will most regularly forage and travel through (Beekman et al., 2004). Although bees do forage further than this, they will have to travel directly through the landscape in close proximity to the hive, and a smaller buffer confines the landscape metrics.

Seven different landscape metrics were calculated (Table 1), reflecting landscape composition: (1) landscape Shannon Diversity; (2) percentage woodland; (3) percentage agricultural cover; (4) percentage urban/suburban cover; as well as landscape configuration: (5) total edge; (6) mean patch size; and (7) cohesion. Metrics were calculated using the ‘landscapemetrics’ package (https://CRAN.R-project.org/package=landscapemetrics; Hesselbarth et al., 2020) in R (r-project.org). In our analysis, only total edge is used to describe landscape configuration owing to correlations between total edge, mean patch area and cohesion. Total edge was therefore determined to be the most relevant based on the studies of Degen et al. (2016) and Menzel et al. (2019).

**Table 1.** Landscape metrics describing the composition and configuration of landscapes surrounding the 12 apiary sites

	Description	Formula
**Composition**		
Woodland cover (%)	Percentage of woodland cover (broadleaved and coniferous) present in the buffer radius	CA=sum(*A*[Patch_*ij*_])
Agricultural cover (%)	Percentage of agricultural cover in the buffer radius	CA=sum(*A*[Patch_*ij*_])
Urban cover (%)	Percentage of urban cover in the buffer radius	CA=sum(*A*[Patch_*ij*_])
Suburban cover (%)	Percentage of suburban cover in the buffer radius	CA=sum(*A*[Patch_*ij*_])
Shannon's Diversity Index – landscape (*H*)	For each buffer radius, measures richness and evenness of the land covers	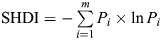
**Configuration**		
Mean patch area (ha)	Mean area of continuous land covers in the buffer radius	*A*_MN_=mean(*A*[Patch_*ij*_])
Total edge (m)	A measure of all edges in the landscape	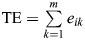
Cohesion	An aggregation metric measuring the physical connectedness of the corresponding patch types	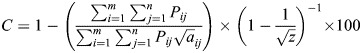

*A* [Patch*_ij_*], area of each patch in hectares; *P_ij_*, perimeter in metres; *a_ij_*, area in square metres; *z*, number of cells; *e_ik_*, edge length in metres; *P_i_* proportion of class *i*.

### Site selection

To select the study sites, the following criteria were applied. Firstly, to reduce variation in habitat types between the landscapes, sites which contained over 75% urban and suburban landscape were not selected. Rural to semi-rural habitats were chosen as substantially more sites fitted these criteria than those heavily dominated by urban and suburban habitat types. This led to the removal of 6 of the initial 26 sites. The minimum distance between sites that were chosen was 1 km to prevent complete overlap of the sites. Two further sites were not used owing to bee colony collapse, which occurred between the time the sites were identified and when the experiment was carried out, leaving 18 possible study sites. To maintain consistency between sites, all hives had been established for at least a year and all had one queen. All hives were successfully producing brood as well as honey and bee bread stores. No hives used were from recent swarms or had recently experienced a change in queen. Additionally, no hives were either currently infected by *Nosema* or had been in the previous 6 months.

One of the primary tasks when considering the study sites was to reduce, or avoid, frequently occurring correlations among the landscape variables, such as between total landscape edge and landscape diversity. To do this Spearman's Rank correlation was calculated between the available sites for landscape diversity and total edge/average patch size (identical selection for each). Cohesion of habitat types against landscape diversity within each 1 km buffer zone was also checked and yielded almost identical results, except for two sites. The selected sites and their characteristics are shown in Fig. S1 and Table S1.

### Free-moving proboscis extension response

To analyse the cognitive responses of bees we used the FMPER method developed by Muth et al. (2018) in the field, measuring aspects of cognition in a similar way to those tested in harnessed bees in the laboratory using proboscis extension response (PER) methods. The FMPER method avoids the negative effects of harnessing, accounts for the differences in behaviour displayed by harnessed versus free-moving bees (Muth et al., 2018), provides ease for the bees to be released after the experiment and is less time consuming, and hence can generate larger sample sizes.

From each hive, 15 foraging active bees were collected for each set of trials in each apiary. Bees were collected by blocking the entrance to the hive and forager bees identified via the presence of pollen in their pollen basket. Bees were collected into individual 50 ml sample tubes, in which 32 holes (5 mm diameter) had been evenly placed to allow air flow. Two holes were drilled into the lid end allowing for the colour strips to be inserted. Bees were left stationary in these tubes for 15 min to allow them to acclimatise to this environment. Each bee then experienced the learning trials as described below, which took a total of 2 h, and occurred outside in a shaded and sheltered location at least 20 m from the hives. Two groups of bees were tested each day, the first group were tested in the morning starting at 09.00 h and the second in the afternoon starting at 13.00 h, with each set using clean sample tubes. Bees from the initial set were not released until the second set of bees had been collected. All bees were marked on return to the hive to prevent recapture of the same individual. No more than 15 bees were collected at a time to minimise the time they would be held in the sample tubes. Of the 1080 bees that were captured, 934 survived to the end of the study; only the data from the survivng bees were used.

Each bee experienced two learning trials and five testing trials. The inter-trial interval was 15 min. For the learning trials, each bee was presented with a positive control stimulus (CS+, coloured strip with a 50% sucrose reward) followed by a negative stimulus (CS–, a coloured strip with 10% sodium chloride). The CS+ used was a yellow strip and the CS– a blue strip. The CS+ was always presented first and the bee was allowed to drink from it for 5 s. This was repeated twice and was carried out as an initial teaching phase to provide an opportunity for the bees to learn the associated reward from each colour strip before being tested. For a further five test trials, the bee was presented with the CS+ and the CS– strips simultaneously, the strip the bee fed on first was recorded. A feed was classed as a direct intentional approach to one of the strips to feed/probe via touch. After the bee had sampled either of the two strips, one strip was removed, and the bee allowed to sample the remaining strip to reinforce the negative or positive reward from the remaining strip.

### Statistical analysis

All data analyses were carried out in R (r-project.org). To determine how successful the learning procedure was across all samples at all locations, a difference in the frequency of bees choosing to feed on yellow strips over blue strips was tested using χ^2^ tests. This was carried out for both the first test and the final fifth test.

**Fig. 2.**
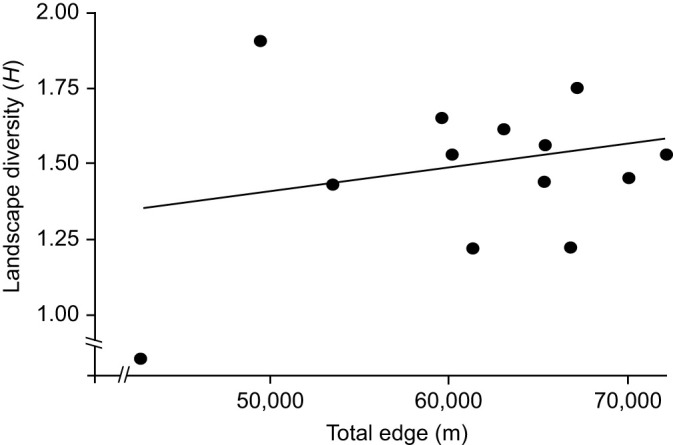
**Spearman correlation coefficients between total edge length and Shannon's landscape diversity (*H*) at the chosen 12 apiary sites.** Little correlation was found between landscape diversity and total edge length (*s*=9,774,066, ρ=0.052, *P*=0.130).

A binomial mixed effects general linear model using the glmer() function from the lme4 package (https://CRAN.R-project.org/package=lme4) in R was carried out to determine if landscape impacted the ability for bees to learn visually using the FMPER. The response was binomial, and where bees chose correctly a ‘1’ was used and incorrectly a ‘0’ during trial 5. Landscape diversity and total edge at a 1 km radius around the hives and the interactions between them were included as fixed factors, whilst date was treated as a random effect. Date was included as a random effect to account for the small weather/seasonal changes across the experimental period. Spearman's rank was used to check correlations between total edge (total edges in metres in the buffer zone) and landscape diversity (Shannon diversity index, *H*), where little correlation was found (*s*=9,774,066, ρ=0.052, *P*=0.130) (Fig. 2). The model used is: Bee learning (trial 5)∼Landscape diversity×Total edge+(1|Date).

## RESULTS

### Honey bee learning

**Fig. 3.**
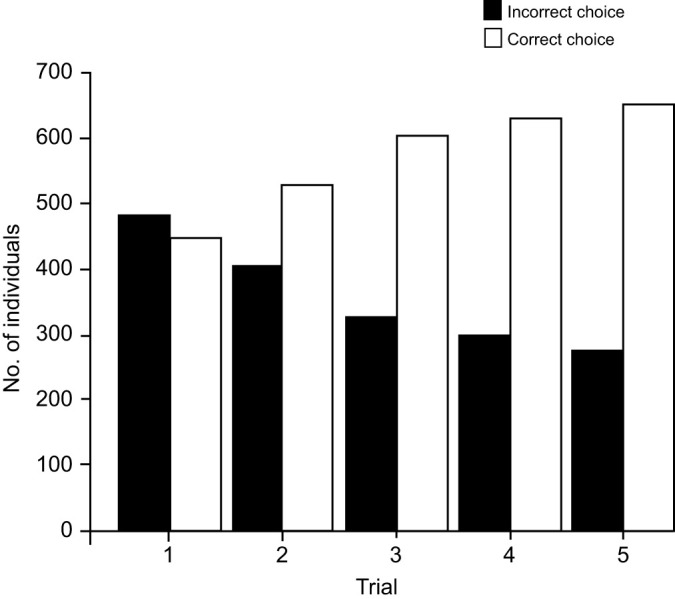
**Honey bees show a conditioned response across five consecutive learning trials.** Learning improved across trails and the greatest number of bees successfully choosing the sugar reward was at trial 5. From trial 2 onwards, significantly more bees chose the positive reward than the negative. Black bars represent the bees that did not learn the conditioned response, and thus chose the blue negative strip containing salt water. Clear bars represent bees that learned the conditioned response, and thus chose the yellow strips containing the sugar water (*n*=934).

During the visual learning trials, honey bees were presented with a yellow and a blue strip. The bees increasingly chose the positively rewarded strip (yellow) across successive learning trials, where over twice the number the bees chose the sugar reward over the negative strip (Fig. 3). At trial 1 just over 400 of 934 bees correctly chose the yellow sugar reward, less than half the bees tested, whereas by trial 3 over 500 of 934 had chosen correctly, and by trial 5 just over 600 of 934 bees correctly chose the yellow reward. The biggest increase in correctly identifying the positive strip occurred between trials 1 and 2, whereas there was only a small increase between trials 4 and 5, which showed a plateau of learning by this point. During trial 1, there was no statistically significant difference between the number of bees that chose the colour blue and those that chose the colour yellow, showing no stimulus bias between strips. Over the following trials there was a significant preference towards the yellow strip, which contained the positive sugar reward (χ^2^=1.388, d.f.=1, *P*<0.239): at trial 2 (χ^2^=16.998, d.f.=1, *P*<0.001), 3 (χ^2^=83.431, d.f.=1, *P*<0.001), 4 (χ^2^=112.320, d.f.=1, *P*<0.001) and 5 (χ^2^=156.24, d.f.=1, *P*<0.001).

### The effect of habitat edge and landscape diversity on bee learning

**Fig. 4.**
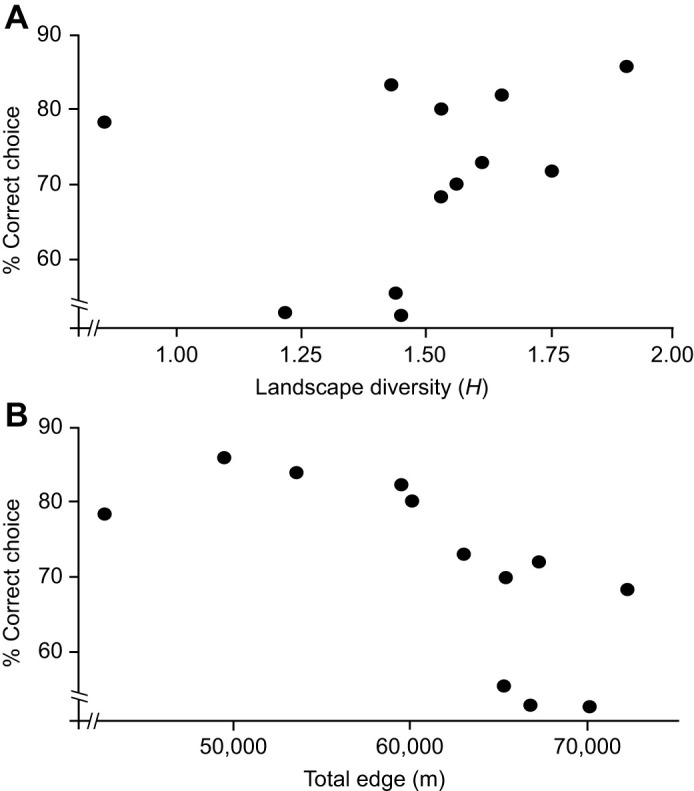
**The effect of landscape diversity and total habitat edge on the percentage of bees choosing the sugar reward (*n*=934).** (A) There was a positive effect of landscape diversity (Shannon's *H*) on the percentage of bees that correctly chose the sugar reward. Bees in landscapes with low total edge made more correct choices compared with bees in landscapes with high total edge.

Between 50 and 80% of bees made the correct choice of the positively rewarded strip across a gradient of different landscape complexities and compositions. Both total habitat edge and landscape diversity were found to affect the learning ability of honey bees. There was a positive effect of landscape diversity on the ability for individual honey bees to learn by the fifth trial (*z*=−4.221, d.f.=929, *P*<0.001). At higher diversity levels of ∼1.75 (*H*), over 80% of bees chose the rewarded strip, but this reduced to ∼50% at a landscape diversity level of 1.25 (*H*) (Fig. 4A). One site did not follow this trend. Here, at a low landscape density of almost 0 (*H*), just less than 80% of bees chose the rewarded strip (Fig. 4A). Bees learned better in areas with less habitat edge (edge *z*=−61.914, d.f.=929, *P*≤0.001) than bees whose hives were situated in an area of more edges. As habitat edge increased, learning ability decreased. When total edge was between 42,000 and 60,000 m per 3.1 km^2^ (13,546 and 19,354 per km^2^) almost 80% of bees chose the rewarded strip at trial 5, whereas this value decreased to 50–70% for edge values between 64,000 and 74,000 m per 3.1 km^2^ (20,645 and 23.870 per km^2^) (Fig. 4B).

## DISCUSSION

Foraging bees are able to navigate in complex environments to locate nectar and pollen sources (Schultheiss et al., 2017). The efficiency of bees in learning and remembering these food sources contributes to overall colony fitness (Raine and Chittka, 2008). Vision is one of the most important sensory modalities for organisms to perceive environmental information, and there is ample evidence of honey bees learning colours, shapes and patterns (Schultheiss et al., 2017), including the ability to discriminate complex forest scenes (Dyer et al., 2008). We found that the landscape within a 1 km radius of honey bee hives impacts visual learning ability in honey bees: colonies situated in landscapes containing fewer habitat edges were able to learn colours better than colonies placed in areas with greater edges.

Whilst this study focuses on associative memory as a first step in understanding the role of landscape on behaviour, it is not only associative memory in isolation that has the potential to be impacted. Navigational learning/memory may also be influenced, not only independently, but also in relation to impacts on associative learning. Honey bees have been shown to use associative grouping and recall of visual stimuli to retrieve appropriate navigational information from memory (Zhang et al., 1999). For example, olfactory cues trigger visual and navigational memories associated with a food site in honey bees, which assists their return to the site (Reinhard et al., 2004). Additionally, although at the neural level very little is known about navigational memory in insects, the mushroom body (MB) has long been shown to be involved in associative memory (Menzel, 2014). More recent studies have also suggested a role of the MB in navigational memory through computer modelling (Ardin et al., 2016). To further develop our understanding of the effects of landscapes on honey bee cognition, landscape navigational memory should also be investigated.

Our results show that honey bees learned to associate a positive sugar reward and negative salt stimulus with a coloured strip over successive trials in the field, with over 70% of bees selecting the positive reward, by the 5th trial. This is in line with results found by Muth et al. (2018) where the percentage of bees choosing the correct colour varied between 0.75 and 0.95 at trial 5 depending on the difficulty of colour discrimination and adverseness of the unconditioned stimulus. Other studies focusing on visual learning through the responses of bees to colour stimuli found that 60% of bees were able to learn the conditioned response (Dobrin and Fahrbach, 2012), whilst 80% of bees are able to recognise facial cues (Dyer et al., 2005). Previous studies investigating olfactory learning in honey bees found similar levels of learning, where 70–80% of control bees were able to learn through the PER (Behrends and Scheiner, 2009; Giurfa and Sandoz, 2012; Matsumoto et al., 2012) and around 50% for SER (sting extension reflex) techniques (Shepherd et al., 2019; Vergoz et al., 2007). Importantly, during the first test trial, bees showed no preference for either the blue or yellow strip, even after the initial two learning trials, indicating that the responses of the bees were not pre-determined to favour either colour strip based on recent foraging experience.

Landscape complexity (total habitat edge) and composition (landscape diversity) were found to affect the learning ability of honey bees. The total number of habitat edges within 1 km of a bee hive was shown to affect the learning ability of a honey bee. Our model showed that fewer edges resulted in greater visual learning per individual honey bee, and thus a simpler landscape may host colonies with greater learning capabilities. This outcome could be explained in several ways. The relationship of a landmark to the goal (e.g. food source) is significant in terms of both distance and specific orientation of the order of appearance between the goal and the landmark (Chittka et al., 1995; Huber et al., 1994). In a complex landscape increased edges and boundaries between landscape types may make a goal conspicuous in comparison to landmark features. In a simple landscape of continuous habitat, however, a goal may be relatively inconspicuous when compared with landmarks in the surrounding landscape and thus make visual learning of these landmarks more difficult and result in bees which are better learners. Alternatively, pollen foraging distances have been shown to be greater in simple landscapes in comparison to complex ones (Steffan-Dewenter and Kuhn, 2003), which may be due to lower resource availability, and thus colonies surrounded by continuous landscape with fewer edges may need to forage further. This may require the bees to be able to learn a greater number of landmarks in order to make a foraging journey.

It is also important to consider that bees do not rely solely on landscape cues to navigate (Vladusich et al., 2005). Bees on a foraging flight may travel several kilometres to visit their goal, and in doing so visit multiple different locations. To conduct these flights efficiently bees must rely on multiple methods. These include visual odometry where the bee estimates the distance travelled by the flow of spatial information sensed by the visual system as well as landmark cues (Dyer et al., 2008). While landscape cues are used across short distances, odometry is also used as a long-range cue (Vladusich et al., 2005). Bee odometry does not rely on colour, using exclusively the signal from their green receptors for determining image velocity (Chittka and Tautz, 2003). Rather than colour, it is the level of intensity contrast present in the landscape that influences the bees estimation of distance (Chittka, 2004). The visual properties of land terrain can vary considerably, depending on both the nature of the vegetation as well as the existence of man-made structures such as pavements or roads (Tautz et al., 2004), and therefore the accuracy and reliance a bee has on visual odometry will vary depending on the landscape. It is possible that in simple landscapes consisting of continuous habitat and few edges that visual odometry is less useful owing to fewer changes in contrast across the terrain, and thus landscape cues become more important in helping bees to navigate to food sources, making them better visual learners. This idea, however, may be contradicted by the result that honey bees surrounded by a higher landscape diversity were able to learn at a higher success rate than bees whose hives were surrounded by less diversity. An increase in landscape diversity will lead to more contrast across the environment and this greater variety in land terrain may lead to bees relying more on odometry techniques and thus result in reduced learning abilities. This, however, was not observed. While changes in landscape diversity were kept to a minimum, an effect was still found, suggesting that landscape diversity may have an impact on visual learning and cognitive ability in the honey bee.

With respect to landscape diversity, it is important to bear in mind the potential secondary effects of the surrounding landscape. For example, malnutrition is a major stressor for bees, and nutritional stress will be experienced when sub-optimal plants are available for foraging (Vaudo et al., 2015). Malnutrition can have effects on learning: when bees were exposed to a pathogen and a low-quality pollen diet, they suffered reduced learning abilities compared with those just infected by the pathogen (Gage et al., 2020). It is possible that bees whose hives were located in a landscape of higher diversity had access to a wider range of nutritional resources from the surrounding environment, resulting in more reliable food supply, a more diverse diet, and enhanced fitness and learning capabilities. In addition, other factors such as phytosanitary treatments, exposure to diesel fumes and other environmental pollutants, such as proximity to powerlines, which have been shown to affect honey bee cognition may vary within the landscapes surrounding the hives (Frost et al., 2013; Girling et al., 2013; Shepherd et al., 2019; Siviter et al., 2018; Williamson and Wright, 2013).

There is also genetic variability between hives and individuals to consider. This includes differential sugar response thresholds, whereby some foragers will be more driven by sugar rewards and will, therefore, be more likely to learn in an experiment (Pankiw and Page, 2000) as well as differences in latent inhibition (Ferdenzi et al., 2014) and reversal learning (Chandra et al., 2000), which again have the potential to impact their learning ability. In the future, it would be beneficial to sample an increased number of hives at particular sites in order to consider these differences in greater detail.

Furthermore, owing to the availability of sites and the requirements that needed to be met by each site (habitat type, hive health and distance between sites), the range in total edge and landscape diversities was similar (see Fig. S1 and Table S1). In future studies, it would be preferable to maintain either total edge or landscape diversity whilst allowing for the maximum variation in the other. This would allow further investigation into how the difference elements of landscape complexity can impact honey bee learning.

The landscape surrounding the hives of honey bees impacts their ability to learn visually: we found that colonies situated in landscapes containing fewer habitat edges were able to learn colours better than colonies placed in areas of greater edges. This is of importance owing to the relationship between colony health and the ability for bees to learn. Bees must learn foraging routes, find profitable flowers, develop spatial maps as well as recognise intruders. If their cognitive abilities are reduced and they are unable to carry out these tasks, this will be detrimental for the continuous development of the colony. These impacts of landscape on the ability to learn may be due to the need to learn and identify inconspicuous landmarks in areas of continuous habitat or a more limited use of visual odometry in these landscapes.

## Supplementary Material

10.1242/jexbio.250057_sup1 Supplementary information
