# Supplementary material for: The impact of landscape complexity and composition on honey bee visual learning
Source: J Exp Biol. 2025 Jul 4;228(13):jeb250057. doi: 10.1242/jeb.250057 (PMC12268175; doi:10.1242/jeb.250057)
Supplement: Supplementary information [file jexbio-228-250057-s1.pdf]

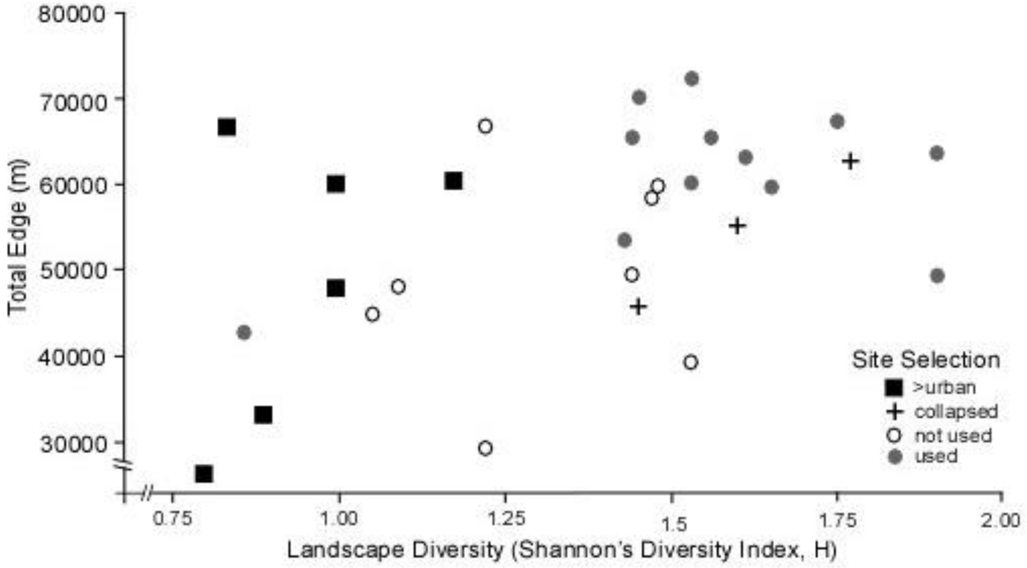

Fig. S1. The total edge length and landscape diversity within the 1 km buffer zones of the 26 potential hives identified in the study. The 12 sites selected to be used are identified by filled circles, while sites that were not chosen are also indicated, including the reason, either the colony collapsed, there was too great a percentage of urban habitat within the area, or the model did not pick them out. Total edge includes all edges within the 1 km buffer (m), landscape diversity uses Shannon's diversity index (H) and is a measure of diversity, taking into account both the number of habitats and their evenness within the landscape.

Journal of Experimental Biology • Supplementary information

Table S1. The landscape diversity, total edge, Agricultural Hectares, Broadleaved Forest, Coniferous Forest, total woodland and Average patch size for the 12 sites used in this today at a 1km radius.

Site N	Landscape Diversity (Shannon)	Total Edge (m)	Agriculture Hectares	Broadleaved Hectares	Coniferous Hectares	Total Woodland Hectares	Average Patch Size Hectares
1	1.56	65400	4.96	70	7.68	77.68	0.987
2	1.43	53540	87.6	24.4	1.84	26.24	1.09
6	1.44	65360	3.32	61	2.04	63.04	0.964
8	1.75	67200	16.7	53.4	83	136.4	0.958
10	1.61	63060	25.9	48.5	2.64	51.14	0.849
13	1.65	59600	38.2	41.5	6.64	48.14	0.914
14	1.9	49420	23.4	13.6	1.88	15.48	1.12
20	1.53	60140	3.64	45	17	62	0.887
22	1.22	66760	1.92	29.9	3.52	33.42	0.932
23	1.45	70080	0.64	54	8.36	62.36	0.806
27	0.857	42780	234	20.6	2.48	23.08	1.34
28	1.24	15860	0.36	5.2	0.08	5.28	1.14

Journal of Experimental Biology • Supplementary information
